# Dynamics simulations of hypoxia inducible factor-1 regulatory network in cancer using formal verification techniques

**DOI:** 10.3389/fmolb.2024.1386930

**Published:** 2024-11-07

**Authors:** Hafiz Muhammad Faraz Azhar, Muhammad Tariq Saeed, Ishrat Jabeen

**Affiliations:** School of Interdisciplinary Engineering and Sciences (SINES), National University of Sciences and Technology (NUST), Islamabad, Pakistan

**Keywords:** hypoxia-inducible factor-1 (HIF-1), vascular endothelial growth factor (VEGF), oglycosylation transferase (OGT), glucose transporter-1 (GLUT-1), extracellular single regulated kinase (ERK), cellular myelocytomatosis oncogene (C-MYC)

## Abstract

Hypoxia-inducible factor-1 (HIF-1) regulates cell growth, protein translation, metabolic pathways and therefore, has been advocated as a promising biological target for the therapeutic interventions against cancer. In general, hyperactivation of HIF-1 in cancer has been associated with increases in the expression of glucose transporter type-1 (GLUT-1) thus, enhancing glucose consumption and hyperactivating metabolic pathways. The collective behavior of GLUT-1 along with previously known key players AKT, OGT, and VEGF is not fully characterized and lacks clarity of how glucose uptake through this pathway (HIF-1) probes the cancer progression. This study uses a Rene Thomas qualitative modeling framework to comprehend the signaling dynamics of HIF-1 and its interlinked proteins, including VEGF, ERK, AKT, GLUT-1, β-catenin, C-MYC, OGT, and p53 to elucidate the regulatory mechanistic of HIF-1 in cancer. Our dynamic model reveals that continuous activation of p53, β-catenin, and AKT in cyclic conditions, leads to oscillations representing homeostasis or a stable recovery state. Any deviation from this cycle results in a cancerous or pathogenic state. The model shows that overexpression of VEGF activates ERK and GLUT-1, leads to more aggressive tumor growth in a cancerous state. Moreover, it is observed that collective modulation of VEGF, ERK, and β-catenin is required for therapeutic intervention because these genes enhance the expression of GLUT-1 and play a significant role in cancer progression and angiogenesis. Additionally, SimBiology simulation unveils dynamic molecular interactions, emphasizing the need for targeted therapeutics to effectively regulate VEGF and ERK concentrations to modulate cancer cell proliferation.

## 1 Introduction

Cancer cell growth is a fundamental biological process characterized by cell proliferation, and an increase in cellular mass typically occurs under stress conditions. This complex process is strictly regulated and relies on vital nutrients like lipids and nucleic acids to provide sufficient energy to synthesize new cellular components. Particularly, cells have developed and improved their mechanisms for survival, allowing them to grow and develop in harsh conditions, which include reduced oxygen levels, inadequate availability of nutrients, and limited energy supplies (Semenza, 2003). However, Hypoxia-Inducible Factor-1 (HIF-1) is a transcription factor widely distributed and expressed in multiple organs in both animal and human organisms. It is crucial in coordinating many physiological reactions to hypoxic circumstances, such as initiating glycolysis to reduce oxygen deprivation and facilitating angiogenesis (Yang et al., 2021). Moreover, the HIF-1 is a central coordinator of many extracellular and intracellular signals associated with cellular proliferation and impacts metabolic control. The multidimensional nature of this entity makes it essential for both innate and adaptive immune responses, highlighting its huge influence on various cellular processes (Ruas et al., 2002).

Dysregulation of the HIF-1 pathway disrupts oxygen balance and growth homeostasis, leading to pathogenic conditions such as cancer, angiogenesis, and metabolic disorders (Semenza, 2010). To pharmacologically target HIF-1 for the therapy of related pathogenic diseases, it is necessary first to understand the coordinated dynamics of HIF-1 regulation (Semenza, 2003), (Boehme et al., 2021). Under hypoxic conditions, the interaction between energy deprivation and restricted nutrition availability occurs through complex mechanisms, impacting transcriptional and post-translational processes at various levels. The changes in gene expression facilitated by HIF-1 initiate disruption in the oxidative metabolism of mitochondria, glucose intake, energy production, and angiogenesis triggering. This process promotes cancer cell migration, survival, and multiplication (et Nurbubu, 2020).

Furthermore, the insulin and Glucagon ratio is important for glucose homeostasis. Low glucose levels under different physiological conditions produced by food intake or stress tumor microenvironments such as oxygen deficiency and inflammation release glucagon from the pancreas to increase blood glucose levels (Melillo, 2006). However, within the domain of cellular signaling pathways, a deficiency in oxygen and essential nutrients can lead to increased levels of reactive oxygen species (ROS). The increase in ROS levels results in the occurrence of oxidative stress, consequently disrupting many signaling pathways (Boehme et al., 2021), (Panieri and Santoro, 2016). It is important to highlight that the actions of HIF-1 and Glucose transporter-1(GLUT-1), which are affected by many types of reactive oxygen species (ROS), give rise to several intracellular effects that facilitate the growth and spread of cancer cells in situations that involve oxidative stress, limited nutritional availability, and energy insufficiency (Nagy et al., 2019).

The complex relationships between HIF-1, O-glycosylation transferase (OGT), Vascular endothelial growth factor (VEGF), and protein kinase B (AKT) are crucial because they all work together to control the expression of several enzymes involved in glucose and fatty acid metabolism as well as the regulation of glucose transporters during the start and development of cancer (Panieri and Santoro, 2016). However, precise metabolic regulation and the best possible functioning of the Mitogen-activated protein kinases (MAPK), AMP-activated protein kinase (AMPK), and AKT/PI3K pathways are necessary to maintain glucose homeostasis, which makes them desirable targets for therapeutic intervention (Jin et al., 2020), (Lee et al., 2019). In this context, HIF-1 plays a crucial role in allowing cells to adapt to hypoxic conditions by coordinating the expression of genes involved in critical processes such as angiogenesis and glucose metabolism via key cancer-related signaling pathways. HIF-1 can promote its cancer-related actions by either acting as a facilitator of oncogenic processes or modulating downstream targets within tumor-promoting signaling pathways (Huang and Zhou, 2020). Although the role of HIF-1 in cancer is well-known, there remains a lack of comprehensive investigation into its connections with important variables such as VEGF, GLUT-1, and OGT within the framework of epigenetic mechanisms, along with other crucial entities such Extracellular single regulated kinase (ERK), β-catenin, p53, AKT, and C-MYC. A thorough examination of these factors is necessary to gain a comprehensive understanding of how they collectively induce the activation of HIF-1 and promote the metastatic progression of cancer.

**FIGURE 1:**
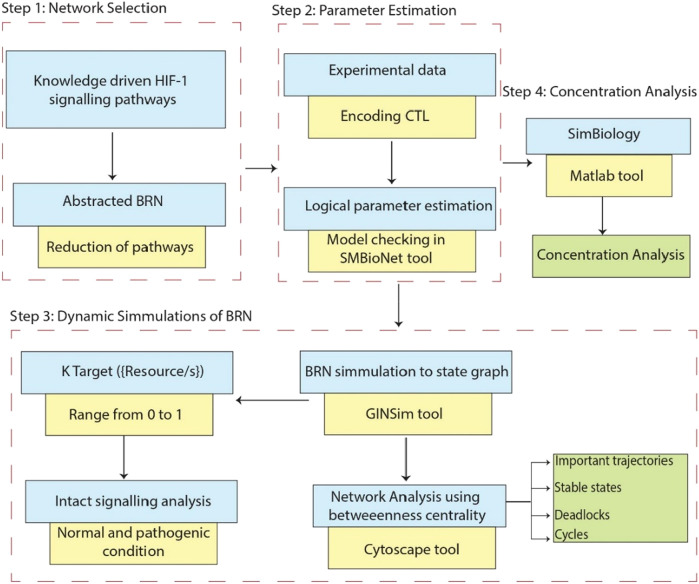
This workflow demonstrates the comprehensive research methodology employed in this study. Step 1: Represents the extraction of the knowledge driven HIF-1 signaling pathways from KEGG database and creating interaction graphs from experimental data to construct a BRN by using reduction rules. Step 2: Computation Tree Logic (CTL) formula is generated based on experimental data and parameters are assigned using SMBioNet, it uses NuSMV for model checking to verify the experimental observation in (Supplementary Material S1). Step 3: Shows the methodology steps for the dynamics simulation of the BRN using the GINSim tool, generating state graphs for the analysis of normal and pathogenic conditions. The resulting state graphs are visualized in Cytoscape, emphasizing the maximum betweenness centrality of states to analyze and identify crucial trajectories, cycles and deadlock states. Step 4: Represents the flow chart of concentration analysis of finally selected entities in SimBiology using Matlab*.*

Prior studies have examined the therapeutic potential of HIF-1 using *in vivo* and computational analyses (Huang and Zhou, 2020), (Poon et al., 2009), (Onnis et al., 2009). However, more research is still needed into their impact on downstream target genes, such as GLUT-1 and the crucially related signaling networks. Consequently, to shed light on the epigenetic regulation of GLUT-1, a crucial component in the initiation and spread of cancer, our research focuses on constructing a Boolean Regulatory Network (BRN) model for HIF-1 signaling. In order to accomplish this objective, we implemented a qualitative modelling methodology that relied on the René Thomas formalism (Thieffry and Thomas, 1995). The wet laboratory data was employed in our study, which was represented using computation tree logic (CTL) to estimate parameters. Additionally, we implemented the model verification technique, as depicted in Figure 1. The primary objective of our comprehensive examination of the model trajectories was to clarify the interconnected pathways associated with the increased expression of HIF-1, the initiation of the oncogene C-MYC, and the suppression of the tumor suppressor gene p53. These elements collectively lead to either the progression or recovery of cancer invasion and homeostasis.

## 2 Materials and methods

The research method employed in this study is visually shown in Figure 1. It describes the procedures followed to complete the study and get the results.

### 2.1 Network selection and theoretical framework for qualitative modeling

In this study, we employ a mathematical framework called the kinetic logical formalism, initially developed by Rene Thomas and refined in prior research on modelling biological regulatory networks (BRNs) (Thieffry and Thomas, 1995). To conduct our modelling, we use the GINsim utility, as described in (Ahmad et al., 2012). However, positive and negative feedback loops are two essential feedback mechanisms in BRNs that kinetic modeling considers and incorporates. The activation of different components within the network is enhanced by positive feedback processes, which are essential in producing several stable states. Conversely, negative feedback mechanisms prevent various components from acting independently and are essential for generating oscillatory behavior or preserving homeostasis, the state of stability within a system (Thomas et al., 1981). Several studies have investigated genetic networks, focusing on formal methods for analyzing positive and negative feedback loops, as first described by (Thieffry and Thomas, 1995).

#### 2.1.1 Reducing signaling pathways

**FIGURE 2:**
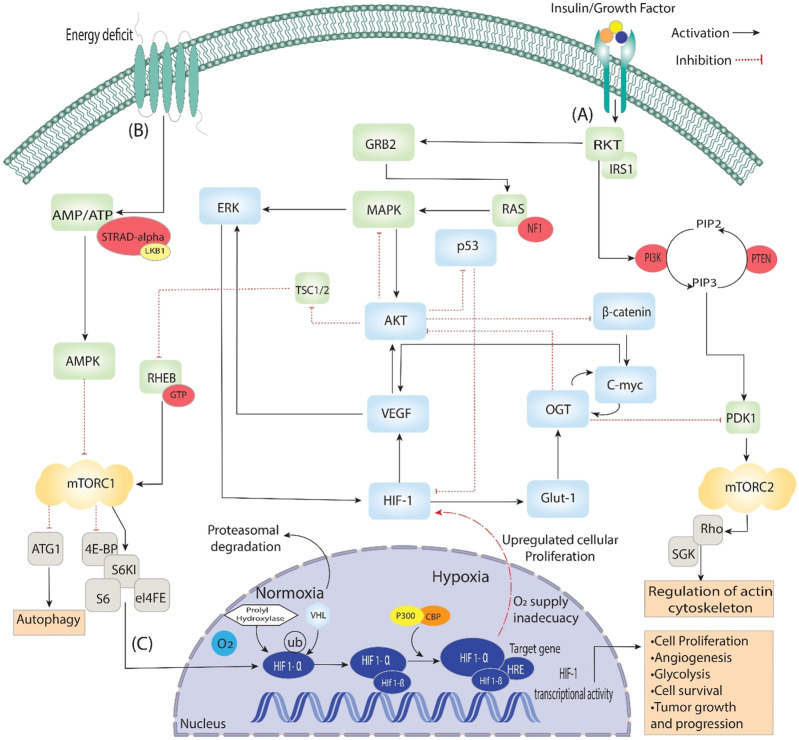
Activating insulin growth factors and energy deprivation triggers the HIF signaling pathway. It is divided into three steps: **(A)** When insulin binds with the epidermal growth factor, it starts signal transduction through auto-phosphorylation. It phosphorylates the ongoing genes RKT, GRB2, IRS1 and PI3k, which converts PIP2 to PIP3, then phosphorylation of PDK1. Additionally, PDK1 phosphorylates mTORC2, which is involved in regulating actin cytoskeleton. On the other hand, GRB2 phosphorylates RAS and activates the MAPK signaling pathway, which starts the phosphorylation of AKT and ERK, directly involved in Angiogenesis through VEGF and HIF-1. Furthermore, AKT is a central pathway regulator that inhibits β-catenin, p53 and TSC1/2, which dephosphorylate RHEB and, through GTPase, activate mTORC1. **(B)** During the energy deficit condition, the AMP/ATP ratio is maintained through AMPK, which dephosphorylates the mTORC1 and is involved in the process of autophagy and activates HIFs. **(C)** In the nucleus, HIF-1 stabilizes, binds VHL/PHD HIF1-α and reaches the target gene with the help of P300, which acts as a coactivator. The HIF-1α/HIF-1β complex binds to the HRE region of the target gene, triggering the hypoxia response and activating various up-signaling genes, including VEGF and GLUT-1, and they activate associated genes.

**FIGURE 3:**
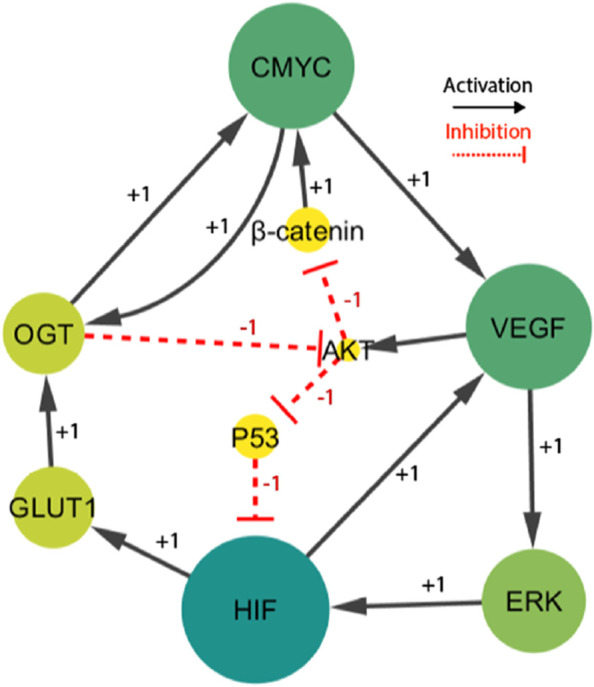
The HIF- one linked BRN reduces the signaling cascade highlighted in Figure 1. Particularly, the sign “+1”indicates a positive (activating) interaction, while the sign '-1′ indicates a negative (inhibiting) interaction.

In qualitative models, the state space of these models grows exponentially with the number of components. A reduction approach has been created to tackle this problem; it seeks to reduce the size of qualitative models while maintaining their structural and dynamic properties. A signaling route is depicted in Figure 2 that has been reduced to produce a Boolean Regulatory Network (BRN) (Cui et al., 2010), (Saeed et al., 2016), as shown in Figure 3. The reduction procedure was carried out by implementing particular reduction rules comprehensively defined in prior research studies (Saadatpour and Methods, 2023), (Naldi et al., 2010). These reduction principles have been used to simplify complex signaling pathways like mTOR, PI3k, and AKT, ultimately creating BRNs that can include any possible regulatory feedback circuits (Saeed et al., 2016).

#### 2.1.2 Semantics of qualitative models

This section provides a brief description of the formal semantics that have been embedded into Rene Thomas' framework (Bernot et al., 2007). The framework presented in this study serves as a foundational theoretical basis for understanding the precise mathematical constructs and principles that govern the intricate dynamics and interactions of components within complex systems. Its application is particularly focused on the domain of biological regulatory networks. The framework developed by Thomas offers a systematic and precisely specified approach for identifying and studying the dynamic behaviors shown by these systems. As a result, it serves as a fundamental basis upon which further research and modelling efforts are constructed.

#### 2.1.3 Definition 1 (BRN)

Biological Regulatory Networks(BRNs), defined as complex systems governing biological processes within living organisms, serve as systematic frameworks for formally representing biological interactions through directed graphs (G = (V, I)). In this representation, 'V' signifies the discrete nodes that comprise a biological system. The set I, which is a subset of V × V, serves to define the interactions between entities, indicating the entities that produce impact on others. Every biological entity inside the network possesses both successors (G^+^vi) and predecessors (G^−^vi) (Bernot et al., 2007).

In this system, every node referred to as an entity is allocated a parameter denoted as ℓvi, which is dependent on the count of its successors. When an entity possesses one or more successors, the value of ℓvi is equivalent to its number of successors. Conversely, if the entity has no successors, the value of ℓvi is equal to 1. Pairs represent the edges present in the network (τ, σ), where τ indicates the type of interaction, denoted by a positive symbol “+” for activation or a negative symbol “-” for inhibition. The influence threshold is defined as τ ≤ ℓvx, which is determined by the specific properties of the interaction τ (Bernot et al., 2007).

A biological regulatory network’s “state” refers to the particular configurations and degrees of expression of each biological component at any one time, providing information about the network’s dynamic behavior and reactions. This information is critical for understanding the network’s functionality as it responds to various stimuli and environmental changes. The provided information offers a concise representation of the behavior and interaction of the components inside the system, hence providing significant insights into its dynamic behavior.

#### 2.1.4 Definition 2 (qualitative state)

The qualitative state of biological regulatory networks can be accurately characterized by the vector (E = ev1, ev2, …, evn), which provides a careful representation of the features of the network components. The comprehensive depiction facilitates understanding the network’s dynamics and adaptive reactions. Each component, evi, correctly denotes the level of expression of a given entity vi and is directly linked to the complete set Ivi. At its core, Evi functions as an abstract concept representing the intricate and situation-dependent expression of the biological entity vi in a certain condition (Bernot et al., 2004). The computation of the state space in a biological regulatory network is a crucial task logically achieved by applying Equation 1. This mathematical equation, similar to a Cartesian product, facilitates a systematic investigation of the extensive and complicated network, which contains huge possible combinations of entities at different expression levels.

$$Z=\prod_{i=1}^{n}Ivi$$ (1)

The letter Z plays a vital role in this equation, representing the vast range of possible network states. It can only be calculated by systematically summing up the many levels of expression that define each entity. Importantly, it must be emphasized that the dynamic interactions with its predecessors intrinsically govern the vi expression level. The complex network of interdependencies among entities and their surrounding variables has a significant role in modeling the diverse range of possible states within the system, illustrating the intricate complexity commonly observed in biological systems.

#### 2.1.5 Definition 3 (resources)

In a biological regulatory network (BRN), represented as G = (V, I), an important concept is introduced: “resources.” The set Rvz is defined as the collection of particular elements vy that belong to the set G, except the element vz and the selection of these elements depends upon specific criteria. Specifically, if Evy is equal to or larger than a certain threshold τvy, vz, and a positive interaction exist shown by αvy, vz = “+,” these elements are chosen. Conversely, if Evy is less than the threshold τvy, vz, and αvy, vz = indicates a negative interaction “-,” these elements are selected. Rvz functions as a repository of resources, while vz symbolizes a variable belonging to the V set, with its state indicated as Evz. The existence of activators and the lack of inhibitors determine whether or not to treat entity “vi” as a resource. If vi has activators, it can be considered a component of Rvi. On the contrary, when inhibitors are present, they have a role in preventing vi creation. As Vi progresses along its evolutionary path toward its goal state, its expression level, represented by Kvi, is closely related to positive integers. These numbers are also known as logical parameters and are grouped under Rvi (Bernot et al., 2004).

The resource idea outlined above is crucial in understanding the complex relationships between nodes in a BRN. These interactions determine the presence or absence of particular activators and inhibitors in addition to the quantitative expression levels of these entities. The complex dynamics of the system are carefully controlled by logical parameters, which significantly impact the network’s behavior and ability to achieve specified regulatory results.

#### 2.1.6 Definition 4 (state graph)

In the graphical representation of a BRN shown as G = (V, I), the state graph, denoted by E = (S, T), includes two important nodes (Bernot et al., 2004).

##### 2.1.6.1 Set of states (S)

This part is a grouping of states, where each state is an arrangement of unique entities in the network. It essentially records the system’s configuration at a certain moment in time.

##### 2.1.6.2 Transition relation (T ⊆ S × S)

This relation describes how the system can change from one state to another and defines the relationship between states. This elucidates the dynamic nature of the network.

The transition relation (T) follows a certain condition: s⇒ s' ∈ T if the following requirements are satisfied.

• In a biological regulatory network, differences in the expression levels of two separate entities, called Evx and Evx', in different states (s and s'), are written as Evx = Evx △ Kx (Rvx).
• Expression levels in state s and state s' are the same for all other entities y ∈ V (apart from x), shown as {x}Epy = Epy.

As a result of this description, we can see how the state graph is built and how changes in the expression levels of certain entities in the network determine transitions between states. The framework offers a systematic approach to understanding the network’s dynamic behavior and the factors that drive its transitions between various states.

### 2.2 Model verification for the parameter estimate

The parameter selection in qualitative modeling of Thomas networks is significantly influenced by logical factors determining the system’s behavior that are initially unknown. The previously mentioned features or parameters play a crucial role in developing the system’s dynamic phenomena such as cycles, stable states and transitions, which are visually represented as a directed state graph (Bernot et al., 2004). The presented graph comprehensively represents significant phenomena to systematically identify and validate these parameters, by employing a formal model-checking approach using the tool SMBioNet, specifically designed to analyze such networks (Khalis et al., 2003). The SMBioNet tool enables the implementation of this method, which is intended to explore various states of the model and match parameters with experimental data ensuring both logical consistency and biological relevance. Parameters that are logically consistent are of utmost significance in qualitative modeling (Thieffry and Thomas, 1995).

In qualitative modeling, parameters do not take numerical values but are instead logical in nature, defining whether a biological entity, such as a gene or protein, is active, inhibited, or neutral. SMBioNet constructed based on qualitative formalism developed by Rene Thomas relies on two phases: the initial phase of the parameter derivation process involves formulating hypotheses based on biological data. This information is encapsulated in a CTL equation that defines temporal properties of biological entities like stability, oscillations and their network interactions, or specific sequences of activation and inhibition expected in biological systems (Baltazar et al., 2011). The second phase set of logical constraints derived from an iterative process to fine-tune the model parameters. These parameters are systematically modified to either satisfy the CTL formulae or match the observed biological behaviors, ensuring logical consistency across different states and conditions within the network (Thieffry and Thomas, 1995), (Baltazar et al., 2011).

However, we used model checking after this iterative change to confirm the settings. The chosen parameters created the intended qualitative characteristics, such as stable states or oscillating patterns, and were consistent with experimental data, SMBioNet thoroughly examined every state of the network (Khalis et al., 2003). The resulting model visually depicted these dynamic behaviors as a directed state graph, emphasizing important characteristics like stable states and cycles essential for comprehending the system’s dynamics.

#### 2.2.1 Quantifiers in computational tree logic

In CTL quantifiers play a crucial role in expressing the properties and behaviors of systems. Quantifiers allow us to explain the existence or absence of certain states or paths inside a system’s state space. However, CTL utilizes a formula (zpi = n) to evaluate the current state. This formula determines whether the expression level of the variable pi precisely matches the required value n. This evaluation process facilitates the systematic analysis of dynamic behaviors in biological systems. The formulation of CTL formulae involves the combination of logical connectives with temporal operators. Within the context of logical connectives, the utilization of negation (“¬”), logical disjunction (“∨”), logical conjunction (“∧”), and implication (“⇒”) is prominent. In addition to this, we incorporate temporal operators that consist of pairs of symbols. In these combinations, the first element may take the form of an “A,” which would indicate the existence of all possible routes, or it could take the form of an “E,” which would indicate the existence of at least one path. After this, operators such as “X” are used to signify the subsequent state, “F” is used to denote any upcoming state, and “G” symbolizes all potential future states. In order to generate formal expressions that specify characteristics and behaviors within a system, the CTL quantifiers and operators are essential components. They provide the accurate and organized expression of system attributes and the need to perform analysis and verification (Bernot et al., 2004).

#### 2.2.2 Definition 5

The construction of CTL formulae, denoted as ϕ, is an essential activity within a Biological Regulatory Network setting described as G = (V, I). The formulae contain atomic expressions that can appear in three unique forms: “⊤,” which represents the Boolean value true, “⊥,” which denotes the Boolean value false, or atomic propositions organized as (vi = n). In this context, the symbol “vi” denotes a variable that is present in the state graph, whereas “n” is a value that lies inside the interval [0, ℓvi]. Furthermore, different logical combinations are observed as atomic expressions in atomic formulae. These logical operators include negation, which expresses the opposite of a condition (¬ϕ); logical conjunction, signifying that both conditions must be true (ϕ ∧ ψ); logical disjunction, indicating that either condition being true is sufficient (ϕ ∨ ψ); and implication, representing that if the first condition is true, it implies the truth of the second (ϕ ⇒ ψ). CTL formulae are instrumental in formally defining the intricate features and behaviors of BRNs by employing these operators to describe and analyse their dynamic interactions. These frameworks offer a strong foundation for representing and examining these networks' dynamic attributes and qualities, enabling a thorough comprehension and detailed investigation of their complex dynamics.

### 2.3 Dynamic simulations of BRN

GINsim, a tool of significant value in biological modelling, is crucial in constructing state graphs that provide insights into two essential dynamics in biological systems. The same phenomenon can be observed through the qualitative cycle represented by the ordered pairs (0,0), (1,0), (1,1), and (0,1). This cycle is a typical reaction mechanism in biological systems to preserve homeostasis. On the other hand, as the state (2,1) illustrates, a qualitative situation may result in various actions, which could eventually lead to pathogenic effects or deadlock states. In particular, four distinct qualitative states collectively constitute a cyclic behavior: (0,0), (1,0), (1,1), and (0,1). These states are crucial in maintaining equilibrium and controlling dynamic processes, making them essential to comprehending the cyclic behavior seen in many biological systems (Schwab et al., 2020).

The development of state graphs, which are representations of the dynamic behaviors observed in biological systems, relies heavily on qualitative modelling methodologies. However, it becomes essential to adopt a systematic method when dealing with a state graph exhibiting higher complexity levels. The complex characteristics inherent in qualitative modelling can result in creating a comprehensive state graph, even when dealing with interaction networks of a relatively small scale. Moreover, the state graphs represent qualitative states using extended binary sequences. Due to the complex structure of these state graphs, conducting a manual analysis becomes difficult. Therefore, researchers frequently employ network analysis techniques based on graph theory to overcome the difficulty of choosing crucial trajectories within state graphs produced by qualitative modelling (Gonzalez et al., 2006). This methodology facilitates a more precise and effective analysis of the complex dynamics essential in biological systems.

#### 2.3.1 Network analysis

Using graph-theoretic approaches has shown to be particularly efficient in examining extensive protein networks. Within the domain of graph connection, network analysis methodologies demonstrate proficiency in structuring the state graph by highlighting nodes according to their betweenness centrality rankings, ranging from the highest to the lowest values. States with high betweenness centralities are of great interest in biological phenomena. These states are more likely to arise and indicate factors that substantially impact numerous biological processes. Utilizing betweenness centrality measures allows for the assessment and differentiation of qualitative states, facilitating the identification of significant and influential paths within a certain cycle (U. B.-J. of mathematical sociology and undefined, 2001). Identifying crucial nodes within complex biological regulatory networks is essential for understanding cellular processes. Centrality, a commonly utilized measure derived from Social Network Analysis in graph-theoretic frameworks, is commonly applied to assign rankings to entities inside these complex networks (Kourtellis et al., 2013). Centrality analysis is a highly important method for identifying crucial aspects and characteristics of complex biological regulation networks. This technique provides insights into the key players and their interactions, enhancing our understanding of these networks.

#### 2.3.2 Definition 6 (quantifying betweenness centrality)

In the framework of state graph analysis designated by “R” betweenness centrality appears as a key metric to assess the importance of particular qualitative states. In order to achieve a more thorough understanding of this concept, it is essential to examine it within the framework of three discrete qualitative states, namely, e1, e2, and e3. These states play a crucial role in comprehensively examining the phenomenon observed in biological systems, highlighting their dynamic behaviors and mechanisms that maintain equilibrium. When attempting to find the most efficient route between states e1 and e2, it is crucial to consider the total number of paths that pass through the intermediate state e3 (L. F.- Sociometry and undefined, 1977).

The betweenness centrality, written as BC(e3), is a quantitative measure that analyses the degree to which the state e3 serves as a bridge or intermediary relationship between other states within the network. The given statement quantifies the proportion of the shortest pathways that include e3 while examining all possible combinations of states between e1 and e2. However, the mathematical representation of e3 by calculating betweenness centrality is described in Equation 2:

$$\text{BC}\left(E_{3}\right)=\sum_{e1\neq e2\neq e3}{BC}^{\left(e1,e2\right)e3}=\sum_{e1\neq e2\neq e3}\frac{\left(\sigma e1,e2\left(e3\right)\right.}{\sigma e1,e2\;}$$ (2)

The symbol σe1, e2(e3) is utilized to quantify the shortest pathways from the initial state s1 to the final state e2 while incorporating the intermediate state e3. It is an effective technique for comprehending and improving systems, especially in domains like network analysis and transportation of genes or entities. Meanwhile, the shortest routes connecting states e1 and e2 are represented by σe1, e2. All possible variations of e1, e2, and e3 states are included in the summing process. It is important to highlight that within the state graph of a biological system, every qualitative state corresponds to a distinct and possible pattern. However, it is important to emphasize that the edges of the state graph are commonly started in random order, which adds to the network’s behavior and structure complexity. This complexity highlights the significance of betweenness centrality to identify the central function of individual states in the larger network dynamics.

### 2.4 SimBiology simulations of solute concentrations

SimBiology, a computational modeling and simulation platform, was employed to investigate the dynamics of solute concentrations within the biological system under study. The model incorporated key biological entities, such as p53, AKT, β-catenin, VEGF, HIF-1, ERK, and GLUT-1, with their initial concentrations derived from literature and empirical data to reflect their typical levels in cancerous tissues. A detailed model of the system was constructed, incorporating the relevant biological processes and interactions. Initial concentrations of all solutes were specified, and the model was simulated over time. The simulated concentrations of various solutes were monitored and analyzed to gain insights into their behavior and the underlying mechanisms influencing their dynamics (David et al., 2015). The simulation was run over biologically relevant time units to capture both rapid signaling events and longer-term equilibrium states. Ordinary differential equations (ODEs) were used to define the interactions between these solutes, with rate constants representing their production, degradation, and feedback mechanisms. These constants were carefully chosen based on experimental data, ensuring the biological relevance of the interactions. Equilibrium concentrations were calculated to identify stable states in the system, and sensitivity analysis was conducted to ensure the robustness of the model, allowing for insights into how small changes in parameters could affect system behavior. Concentration versus time curves were plotted to visualize the changes in solute concentrations over time. Equilibrium concentrations were calculated to identify the stable state of the system. SimBiology built-in functions were utilized to assess concentration gradients, fluxes, and accumulation areas, providing further insights into solute distribution and transport within the system (Bassingthwaighte et al., 2012).

## 3 Results and discussions

### 3.1 Network selection

A knowledge-driven qualitative Biological Regulatory Network (BRN) of HIF-1 was extracted from the literature. The selection of specific entities (HIF-1, ERK, VEGF, p53, β-catenin, AKT, GLUT-1, C-MYC, OGT) for the creation of the BRN is based on their significant contribution to complex cellular processes in cancer. Understanding these entities and their interactions is crucial in unraveling cellular control and investigating new therapeutics linked to cancer. These entities are intertwined within a complex network, where the activation or dysregulation of one entity sets off a chain reaction, influencing others and contributing to cancer progression. Based on the comprehensive signaling pathways shown in Figure 2, HIF-1, a transcription factor sensitive to reduced oxygen levels (hypoxia), initiates the activation of specific genes, including VEGF and GLUT-1. These genes are known to have a significant influence in facilitating angiogenesis and enhancing glucose uptake, respectively. The impact of the activation of VEGF and GLUT-1 is highly critical inside the hypoxic tumor microenvironment, specifically for tumor growth and metastasis. (Maxwell et al., 1997), (Wincewicz et al., 2007). As a result, the activation of ERK, a central regulator in cell signaling, leads to increased cell proliferation and survival, fueling uncontrolled cancer growth (Minet et al., 2000). The p53 protein, a critical factor in suppressing tumor growth, assumes a central position in regulating the cell cycle and programmed cell death, known as apoptosis, to maintain cellular homeostasis. Disruptions in the p53 gene’s functionality impair crucial regulatory processes, hence facilitating uncontrolled cellular proliferation, frequently observed in many human malignancies. It highlights the significant consequence of p53 in inhibiting abnormal cell growth and its involvement in preventing tumors (Sermeus and Michiels, 2011). Due to this, dysregulation of β-catenin within the Wnt signaling pathway disrupts normal cell adhesion and gene expression, thus raising cancer development and AKT’s role in cell survival and proliferation grants cancer cells a survival advantage (Cui et al., 2010). Moreover, the upregulation of GLUT-1 is strongly associated with the Warburg effect, a metabolic phenomenon observed in cancer cells characterized by enhanced glucose uptake (Li et al., 2015). Subsequently, C-MYC, an oncogene, boosts uncontrolled cell division, and OGT’s capacity to modify protein function can significantly impact cellular processes and potentially contribute to cancers (et Nurbubu, 2020). These entities constitute a highly complex and intertwined network, highlighting the significance of comprehending their roles within regulatory networks to unravel disease mechanisms and identify potential therapeutic targets. Qualitative modelling is employed to dissect these entities’ stepwise processes and interactions, providing a detailed understanding of how they influence each other and cellular behavior in cancer.

The finally selected network included fourteen interactions and nine biological entities, as shown in Figure 3. The selected activator entities, including HIF-1, VEGF, and ERK, create a positive feedback loop, which is recognized to yield numerous stable/deadlock states. A positive feedback loop comprises positive interactions and an even number of inhibitory or negative interactions (PLAHTE et al., 2011). It functions similarly to a toggle switch, simultaneously expressing just one of the two entities (Gardner et al., 2000). A negative circuit comprises a negative interaction of an odd number that results in homeostasis or periodic behavior, such as the interaction between C-MYC, VEGF, AKT, and β-catenin. Three complex HIF-1-based regulatory circuits generate cell proliferation and regulate oscillations. The circuits under consideration consist of three distinct positive feedback loops. The first loop involves the interaction between VEGF and ERK. The second loop incorporates VEGF, AKT, and p53. The third loop comprises C-MYC, VEGF, ERK, GLUT 1, and OGT. A comprehensive examination of these regulatory patterns provides useful insights into the various behaviors that the system could show. Moreover, the computation of logical parameters exposes the huge functional dynamics that have both positive/negative routes in a complicated system.

### 3.2 Parameters estimation

Computing logical parameters in biological regulatory networks is crucial for modelling complex systems, predicting network behavior under different conditions, and gaining insights into biological processes. The model trajectories are derived by examining logical parameters' binary values (0 or 1), visually represented as a state transition graph in Figure 5. These parameters are computed by utilizing Computational Temporal Logic (CTL) algorithms, which are driven by well-established qualitative observations (Baltazar et al., 2011).

The CTL data presented in Table 1 shows changes in the HIF-1 route, which is the first formula in the biological system that expresses high expression of VEGF, OGT, and GLUT-1, all of which are required for tumor cell proliferation, vasculogenesis, and metastasis (Chae et al., 2011), (Hendriksen et al., 2009), (Lei et al., 2020). Under hypoxia, hepatocellular carcinoma cells with high HIF-1 and GLUT-1 expression regulate tissue oxygen supply and energy metabolism (Chae et al., 2011), (Hendriksen et al., 2009), (Amann et al., 2009). The downregulation of Akt in hypoxic conditions is a part of the cellular response to adapt to the stress of low oxygen. This downregulation is thought to be a protective mechanism, as it helps to conserve energy and limit processes that require oxygen, such as cell proliferation. Additionally, the downregulation of AKT in hypoxia contributes to the activation of alternative survival pathways that are better suited for low-oxygen environments (Mottet et al., 2003). However, decreased AKT expression owing to dephosphorylating progressively leads (symbolized by “CTL operator ⇒“) to a stable pathogenic/oncogenic state (symbolized by CTL operator EF and AG) for all entities with high expression levels also for one path future state and all paths globally except AKT.

**TABLE 1:** The SMBioNet setup utilizes Computational Temporal Logic (CTL) equations to infer logical parameters. The second formula is specifically constructed to monitor homeostasis, whereas the other two are intended to observe a state of stability or homeostasis.

No.	CTL formula	References
1	((VEGF = 1&OGT = 1&GLUT1 = 1&HIF-1 = 1&AKT = 0&P53 = 1)⇒ EF(AG(VEGF = 1&OGT = 1&GLUT1 = 1&HIF-1 = 1&AKT = 0&P53 = 1)))	Chae et al. (2011); Lei et al. (2020); Sermeus and Michiels (2011)
2	and((VEGF = 0&OGT = 0&GLUT1 = 0&HIF-1 = 0&AKT = 1&P53 = 0)⇒ EF(AG(VEGF = 0&OGT = 0&GLUT1 = 0&HIF-1 = 0&AKT = 1&P53 = 0)))	Mottet et al. (2003)
3	and((VEGF = 0&HIF-1 = 0&GLUT1 = 1&AKT = 1&P53 = 1)⇒ EX(EF(VEGF = 0&GLUT1 = 1&ERK = 1&OGT = 1&CMYC = 1 &BCAT = 1&HIF-1 = 0&AKT = 1&P53 = 1)))	Amann et al. (2009); Chae et al. (2011); Das et al. (2015)

Only those parameters that fulfill the CTL formulae were chosen using the SMBioNet software (Helikar et al., 2011). It lists all the models in detail and picks the ones whose sets of parameters match the experimental data given as temporal logic formulae. As a result, eight sets of logical parameters were generated by SMBioNet, all of which point to a single deadlock state (1,1,1,1,1,0,1,1,1). The source code of SMBioNet and the output results used to calculate model parameters are found in (Supplementary Material S2). The existence or absence of resources determines every entity’s ability to adjust or modify its expression level. The present state of a gene is compared to values of logical parameters indicated in Supplementary Table S1 at each given time instant to determine its change in expression level. According to the calculated values, ERK maintains or increases its expression level in the presence of a HIF-1 activation signal.

In response to ERK activation, a simultaneous upregulation of HIF-1 expression is observed while the inhibitory influence of p53 remains present. Only in the absence of an AKT inhibitor signal does the tumor suppressor protein p53’s expression level rise. The third formula in Table 1 considered as VEGF and HIF-1 expression suppressed while maintaining GLUT-1, p53, and AKT expression levels. The interplay among glucose transporters (GLUTs), hypoxia-inducible factor 1 (HIF-1), and glycolytic enzymes has been observed across a spectrum of cancers (Chae et al., 2011; Amann et al., 2009; Das et al., 2015). However, particularly intriguing is the exploration of outcomes arising from the simultaneous inhibition of HIF-1 and activation of GLUT-1.

**FIGURE 4:**
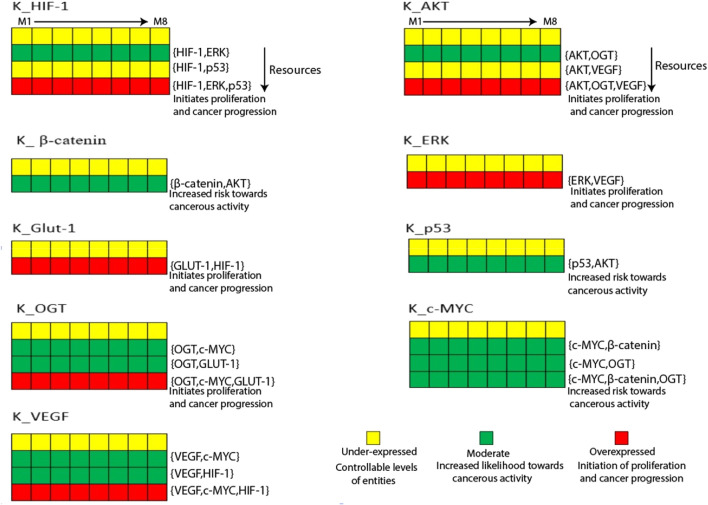
The Heatmap generated from the logical parameters computed on SMBioNet reveals the presence of eight distinct parameter sets. Among these, a preferred set of parameters was determined via model checking, and the results are visually depicted in the form of a heatmap, accompanied by their respective resources labeled as M1 through M8. Each column in this heatmap represents a distinct set of logical parameters, with green denoting moderate expression, red denoting overexpression, and yellow denoting under expression of an entity.

HIF-1 and GLUT-1 regulate mRNA and protein levels in human gastric and ovarian cancers (Amann et al., 2009). State transition graphs are primarily used to examine the mutual behavior of genes functioning in a biological system (Schwab et al., 2020). Figure 4 shows a heatmap of the eight sets of logical parameters SMBioNet created in this scenario. Model validation of these data sets confirms the chosen logical parameters, which offer insights into potential biological pathways associated with cancer invasion and recovery. Briefly, the induction of HIF-1 in the presence of ERK is a constant observation across all eight parameter sets, indicating that a parameter set that permits HIF-1 to reach its maximum threshold value of '1′may be harmful to cancer cells. In maintaining natural dynamic processes, these parameters (M8) allow for interactions across all nodes while maintaining their interdependencies for activation and suppression. The state transition graph represents these dynamics. The source code of the input models can be found in the (Supplementary Material S3). The computed values also show that when GLUT-1, VEGF, OGT, and the oncogene C-MYC upregulate, HIF-1 continues to be expressed at a higher level. On the other hand, activating the p53 inhibitor stops HIF-1 from rising above the typical threshold value. Furthermore, in the absence of AKT inhibitory signals, p53 expression rises, and GLUT-1 is activated in response to the HIF-1 signal (Yeung et al., 2008). Interpreting the trajectories within the state transition graph is essential to thoroughly understanding gene behavior in the context of a dynamic biological system.

### 3.3 Dynamic simulations of BRN

#### 3.3.1 State transition graphs

**FIGURE 5:**
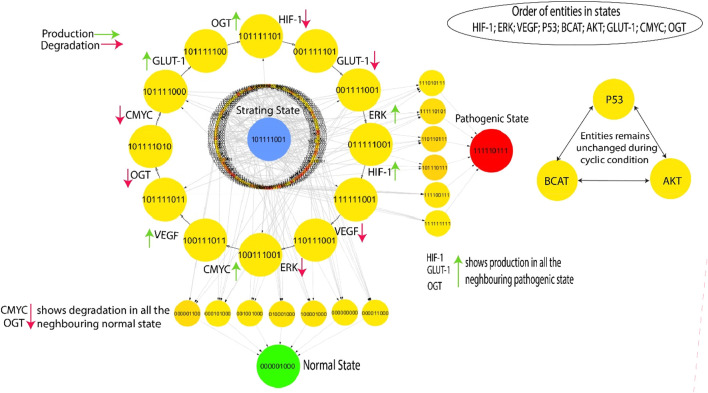
The highest betweenness and centrality cycle is retrieved at the outermost layer. The top layer entities show arrows (red and green) representing a specific entity’s production and degradation. Furthermore, the neighboring entities of the pathogenic state (111,110,111) show continuous activation of HIF, GLUT-1, and OGT, whereas the neighboring entities of the normal state (000,001,000) show continual degradation of C-MYC and OGT.

The Cytoscape software is employed to visualize the state transition graph consisting of 512 nodes (Figure 5). The state graph is created by the GINsim tool using the estimated SMBioNet parameters as indicated in the last column of Supplementary Table S1. The states of biological entities are arranged in a manner that reflects their betweenness centrality. The state graph represents the system’s status at any given time using an indicator containing the expression levels of all entities provided. These entities are ordered: (HIF-1, ERK, VEGF, p53, β-catenin, AKT, GLUT-1, C-MYC, OGT). A typical qualitative condition within the state graph in Figure 5 is distinguished by reduced expression levels of HIF-1, VEGF, ERK, GLUT-1, and OGT as well as the tumor suppressor genes C-MYC, p53. On the other hand, the pathogenic qualitative condition is characterized by increased levels of HIF-1, VEGF, GLUT-1, and ERK expression. This visual depiction facilitates comprehension of the dynamic functioning of the system and offers valuable insights into the shift from a healthy to a diseased state.

This qualitative state (0,0,0,0,0,0,0,0,0) in biological systems exhibits oscillatory behaviors or homeostasis under normal conditions; in a normal state, the overall behavior of the system shows cyclic. As a result, a good qualitative model should include disease/pathogenic and typical homeostatic characteristics such as cycles and closed paths. Under typical physiological conditions, the biological system exhibits homeostasis, defined by the downregulation of oncogenes such as OGT, GLUT-1, and C-MYC. This state is achieved when VEGF activation is absent and reduced levels of ERK. In contrast, a pathological condition characterized by high levels of HIF-1 and oncogene expression is indicated by the trajectory (1,1,1,1,1,0,1,1,1) as illustrated in Figure 5. The transition from homeostasis to a pathogenic state is a significant alteration in the system’s behavior, frequently linked to the progression of the disease. The deadlock state’s mediate predecessors up to two layers have low betweenness centrality, illustrated by circles with significantly greater influence and yellow color. This indicates a greater chance of the infected system regaining stability by passing through a sequence of stable states that hold less importance within the recovery process, as denoted by their low betweenness centrality. However, high betweenness centrality indicates essential nodes that control information flow and influence network dynamics, offering insights into key regulators or potential intervention points in disease mechanisms. The previously mentioned trajectories are crucial in enabling interactions inside the system, significantly enhancing its adaptability and flexibility. The model (Figure 5) highlights a series of cycles computed using the GINsim software, which is shown as the outermost circle. The states within the cycles demonstrate significant levels of betweenness centrality, as denoted by the yellow circles, whereas red circles depict the states with low betweenness centrality. This representation helps us better understand the network structure and how it affects the system’s dynamics.

#### 3.3.2 Selection of cycle

The occurrence of cycles within the model holds significant importance, enabling the finding of the most probable biological cycle. Figure 5 presents a computed cycle utilizing the Cytoscape tool, which uses betweenness centrality to rank states. The ranking of states based on their betweenness centrality is further information in (Supplementary Material S4). In this visual depiction, nodes with larger diameters indicate states with greater betweenness centrality. This methodology facilitates the identification of crucial states within the network, providing insights into the most significant components and their possible effects on biological processes. The cycle with the highest betweenness centrality: (1,0,1,1,1,1,1,0,1) → (0,0,1,1,1,1,1,0,1) → (0,0,1,1,1,1,0,0,1) → (0,1,1,1,1,1,0,0,1) → (1,1,1,1,1,1,0,0,1) → (1,1,0,1,1,1,0,0,1) → (1,0,0,1,1,1,0,0,1) → (1,0,0,1,1,1,0,1,1) → (1,0,1,1,1,1,0,1,1) → (1,0,1,1,1,1,0,1,0) → (1,0,1,1,1,1,0,0,0) → (1,0,1,1,1,1,1,0,0) shows oscillations of all entities except p53, β-catenin and AKT. The cycle indicates that homeostasis requires continual activation of p53, β-catenin and AKT. By disrupting this process, commonly achieved by the overexpression of HIF-1, GLUT-1, and OGT, a transition can occur, leading to either a malignant state (1,1,1,1,1,0,1,1,1) or a state of recovery (0,0,0,0,0,1,0,0,0). This binary sequence appears to represent a state where several signaling pathways and molecules associated with cancer are activated or upregulated. This could indicate a malignant state with increased cell proliferation, survival, and angiogenesis. The “0”for AKT might suggest a potential inhibition or downregulation of this pathway, which is often associated with anti-apoptotic signals. The transition process is made possible through the downregulation of C-MYC and OGT, two crucial factors involved in cellular growth and metabolism (Ferrer et al., 2014), (Jóźwiak et al., 2014). The previously mentioned dynamics highlight the system’s susceptibility to changes in crucial regulatory elements, which affect the balance between normal and disease states.

The cyclic trajectories illustrated in Figure 5 precisely indicate the presence of p53, β-catenin, and AKT proteins. These entities fulfill a useful regulatory function in the hypoxia-inducible factor (HIF) system, aiding in preserving homeostasis within the model. On the other hand, when HIF-1 expression is dysregulated and causes the activation of cellular proliferation mechanisms, particularly ERK and GLUT-1, it has the potential to give rise to problems such as diabetes or more severe illnesses such as oncogenes is. This highlights the crucial significance of these regulatory components in regulating the dynamic equilibrium of the system and its potential deviation toward pathological conditions.

#### 3.3.3 Subgraphs

**FIGURE 6:**
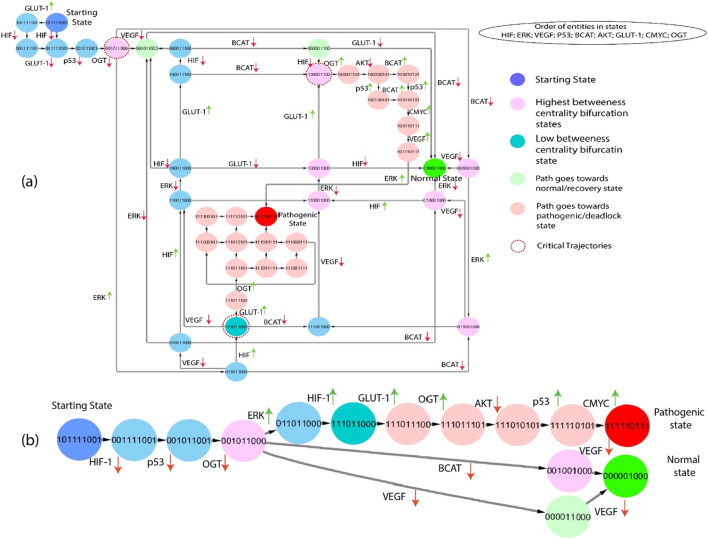
**(A)** The subgraph depicts the entities starting from the state (101,111,001), showing blue and inducing step-by-step changes of the path that goes towards the normal or pathogenic state. Green entities show that the path goes towards the recovery state from different bifurcation points (pink color), and red indicates that the path goes towards the pathogenic state. Moreover, red circles show important critical trajectories at different points. **(B)** This path derived from the network shown in part **(A)**, clearly illustrates the critical trajectories as they transition from the bifurcation point toward normal and pathogenic states.

**FIGURE 7:**
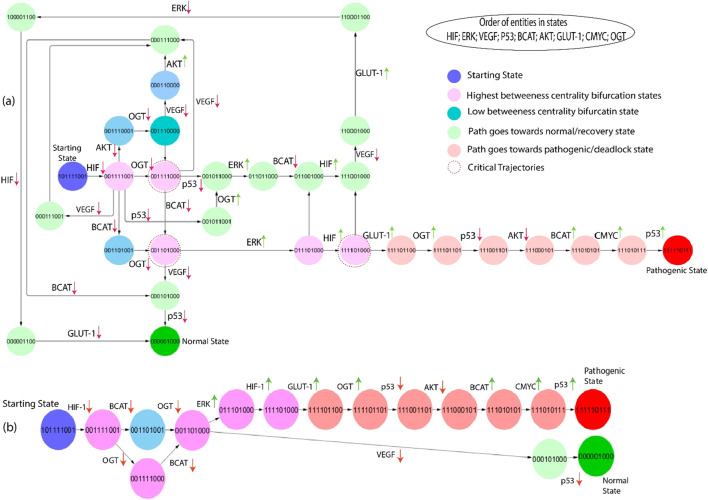
**(A)** The subgraph depicts the same entities as Figure 6, starting from the state (101,111,001) showing blue and inducing step-by-step path changes towards the normal or pathogenic state. Green entities show that the path goes towards the recovery state from different bifurcation points (pink color), and red indicates that the path goes towards the pathogenic state. Moreover, red circles show important critical trajectories at different points. **(B)** This path extracted from the larger network as they transition from the bifurcation point toward normal and pathogenic states.

The subgraphs are extracted from the state graph and shown in Figures 6, 7. These subgraphs depict the behavior of states step by step to induce changes and show how the biochemical system goes toward cancer progression or recovery state. In light of the complex structure of the broad state network that arises from qualitative modelling, we utilized the concept of average betweenness centrality, as outlined in Definition 6, to identify significant trajectories. This methodology enabled us to prioritize significant trajectories without needing to examine each individually, as shown in Figure 6. The chosen trajectory was decided by identifying the maximum betweenness centrality. This trajectory initiated from the initial state (1,0,1,1,1,0,0,1) and led to either the pathogenic or deadlock state (1,1,1,1,1,0,1,1,1). Proto-oncogenes such as C-MYC and AKT are essential constituents of the signaling network under normal physiological circumstances, exerting significant influence on the regulation of cellular development (Hers and Vincent, 2023), (Asano et al., 2004). The previous path selection highlights the importance of these parameters and their possible influence on the shift from a normal to a pathogenic state.

Gain-of-function mutations turn a proto-oncogene into an oncogene, resulting in increased protein expression, signaling pathway alterations, and increased glycolytic flux via the HIF-1 system and inflammatory response. HIF-1, VEGF, p53, β-catenin, AKT, and OGT expression is increased in critical trajectories that begin at (1,0,1,1,1,1,0,0,1). When the body is under stress, increased ERK expression is considered to signify a metabolic transition from oxidative phosphorylation to glycolysis. HIF transcriptional response has been shown to activate in response to this stress, subsequent in the qualitative state (1,1,1,0,1,1,0,0,0). As shown in Figure 6, the following trajectories illustrate that HIF-1 is overexpressed in numerous states, resulting in a pathogenic/deadlock state, validating the increasing evidence that HIF serves various functions during increased cellular metabolism.

#### 3.3.4 Bifurcation states

The dynamic qualitative model illustrates a pathogenic or deadlock condition (1,1,1,1,1,0,1,1,1) represent AKT is inhibited in hypoxic conditions as part of the cellular response to low oxygen, which involves the activation of HIF-1 and changes in energy metabolism. Conversely, AKT is activated in homeostatic conditions (0,0,0,0,0,1,0,0,0) when cells receive signals from growth factors and have sufficient nutrients for growth and survival. The regulation of AKT in different conditions underscores its key role in integrating signals related to oxygen availability, energy status, and cell survival, as evidenced by previous studies highlighting its central role in cellular signaling pathways and its significance in responding to changes in oxygen levels, energy fluctuations, and promoting cell survival (Hoxhaj and Cancer, 2020), (Jóźwiak et al., 2014). The state graph exhibits cyclic patterns of β-catenin, p53, and AKT, specifically oscillations. These patterns indicate the dynamic nature of the system. The presented graphical representation offers valuable insights into the dynamics of state transitions and the functions performed by crucial regulatory entities. The biological system might follow numerous pathways and qualitative states. One of the bifurcation points (1,0,0,0,0,1,1,0,0) goes towards the deadlock state (1,1,1,1,1,0,1,1,1). The sequence of subsequent changes in gene expression determines the precise trajectory of a biological system’s progress toward a goal. For example, prolonged stimulation of VEGF and ERK results in a pathogenic/deadlock state. When a biochemical system reaches a point of no return, it cannot return to a bifurcation point or any other qualitative state and never moves back to the normal homeostatic response. A crucial component of this research entails the computation of logical parameters that exclude the qualitative condition (1,1,1,1,1,0,1,1,1) as the state of a deadlock. The development of a realistic therapeutic objective is of the greatest significance in facilitating the shift of the biological system from a deadlock to a state of recovery or homeostasis. Identifying and subsequently targeting these factors makes it possible to construct therapeutic interventions that efficiently lead the system toward a healthier and more stable state.

#### 3.3.5 Highest betweenness centrality paths

This Qualitative model simulates the interactions between genes and proteins in cancer progression. The paths shown in Figures 6, 7 are extracted from the state graph based on higher betweenness centrality. Figure 6 graph shows that one of the critical trajectories or bifurcation points (0,0,1,0,1,1,0,0,0) goes towards the recovery state if VEGF and β-catenin expression is low. However, if ERK expression is high, the cancer cells will move towards the more aggressive state (0,1,1,0,1,1,0,0,0). The sequence of subsequent changes in gene expression determines the precise trajectory of a biological system’s progress toward a goal. However, in the subsequent critical trajectory, there is an observed increase in the expression levels of HIF-1 and GLUT-1, while the expression levels of β-catenin, VEGF, and ERK are decreased. This leads to the formation of a specific state that includes the expression pattern (1,0,0,0,0,1,1,0,0). The observed transition signifies a noteworthy alteration in the system’s behavior, marked by modifications in the expression levels of crucial regulatory entities. This transition holds potential significance for therapeutic interventions and understanding disease states. At this bifurcation state (1,0,0,0,0,1,1,0,0), HIF-1 expression is downregulated, and the system returns to a normal or homeostatic state despite the elevated expression of AKT and GLUT-1 that are often overexpressed together in cancer cells. This co-overexpression leads to several synergistic effects that promote cancer progression (Gang et al., 2017). Whereas upregulation of OGT with the subsequent consistent activation of AKT and GLUT-1 augment, the hyper-activation of HIF-1 leads to a pathogenic/deadlock state because it creates a positive feedback loop that drives cancer cell growth and survival. Alternatively, the third bifurcation point (1,1,1,0,1,1,0,0,0) illustrates that inhibiting/downregulating VEGF, ERK, GLUT-1, and HIF-1 expression prevents tumors growth and metastasis and promotes a return to a homeostatic state. In contrast, high expression of GLUT-1 leads to a pathogenic/deadlock state. However, the potential therapeutic target is HIF-1 along with VEGF and β-catenin because in the presence of HIF-1, VEGF and β-catenin can work together to promote tumors growth and metastasis (Zhang et al., 2017), (Ye et al., 2018), (Kaidi et al., 2007). Due to the influence of these two entities, GLUT-1 expression is also inhibited, but if HIF-1 and GLUT-1 expression are high along with OGT, the system never returns to normal homeostasis or recovery state. As a result, collectively controlled expression of VEGF, GLUT-1, and OGT to inhibit cancer progression.

The second-largest betweenness centrality path is taken from the state graph to emphasize the significant trajectories. The first bifurcation point (0,0,1,1,1,1,0,0,1) is shown in Figure 7. If VEGF and OGT expression is downregulated from this state, the system returns to a homeostatic or recovery state; when VEGF and OGT expression is downregulated, HIF-1 expression may also be downregulated. This may happen because VEGF and OGT can both stabilize HIF-1 protein levels. By downregulating VEGF and OGT expression, HIF-1 protein levels may decrease, which could lead to a decrease in HIF activity, whereas inhibiting OGT expression moves towards the next bifurcation state (0,0,1,1,1,1,0,0,0). The first critical trajectory (0,0,1,1,1,1,0,0,0) illustrates the same as shown in Figure 6, inhibiting VEGF and β-catenin goes towards recovery state, but if p53 expression in downregulated shows that path goes towards next state. At the same time, β-catenin is downregulated and moves towards the next critical trajectory. The most significant trajectory (0,0,1,1,0,1,0,0,0) shows the expression of VEGF and ERK; VEGF is downregulated simply as the system goes towards a normal or recovery state. The crucial role of ERK at this state shows high expression and moves towards the next Bifurcation state (1,1,1,1,0,1,0,0,0). VEGF can activate ERK, and ERK can activate HIF-1, that are involved in angiogenesis and cell proliferation. This creates a positive feedback loop that can drive tumors growth and metastasis. At this point, p53 is a downregulated path that goes toward another state where initially HIF-1 and GLUT-1 expression is increased, but due to inhibition of VEGF, which leads to downregulation of ERK and ultimately HIF-1 and GLUT-1. However, p53 can inhibit ERK activity, but mutated p53 cannot. This means that cancer cells with mutated p53 will have high levels of ERK activity, which can promote tumor growth and metastasis. However, if upregulation of the HIF-1 signaling from this critical trajectory (1,1,1,1,0,1,0,0,0) causes hyper-activation of GLUT-1 and the path goes toward pathogenic or deadlock state.

When a biochemical system reaches a point of no return, it cannot return to a bifurcation point or any other qualitative state and never moves back to the recovery or normal homeostatic response. Calculating logical parameters is of crucial importance in ensuring that the dynamic models do not include the qualitative state (1,1,1,1,1,0,1,1,1) as the state of deadlock. In hypoxia stabilizing HIF-1α through inhibition of glycogen synthase kinase 3β and prolonged hypoxia leads to AKT inactivation. However, subsequent downregulation of HIF-1 activity by decreasing HIF-1α accumulation, highlighting a biphasic effect on HIF-1α stabilization in response to varying oxygen levels (Mottet et al., 2003). Identifying a realistic therapeutic objective is a crucial step that directs the biological system from a pathogenic condition toward a normal or homeostatic state. Recognizing these factors makes it feasible to create efficient therapeutic strategies for returning the system to a balanced or healthy state.

The fundamental difference between these two biological routes as shown in Figures 6B, 7B lies in their outcomes and implications, particularly in cancer. One of these pathways exhibits a state characterized by a deadlock with elevated expression levels of GLUT-1 and OGT. In contrast, the other pathway demonstrates an increased expression of VEGF and ERK, and importantly, it never reverts to a normal physiological state. In the first route, marked by high GLUT-1 and OGT expression, the cells exhibit a metabolic state often associated with cancer cells. However, GLUT-1 is responsible for glucose uptake, and OGT is involved in adding O-GlcNAc modifications to proteins. The state in inquiry is frequently distinguished by heightened glucose metabolism, referred to as the Warburg effect, which supplies cancer cells with the requisite energy and substrates for accelerated growth and multiplication (Vaupel et al., 2019), (Heiden et al., 2009). This pathway is like a “deadlock” because it reinforces the cancerous phenotype, making it challenging for the cells to revert to their normal, non-cancerous state. Conversely, the second pathway with elevated VEGF and ERK expression is significant for angiogenesis and tumor progression. VEGF is a very influential pro-angiogenic factor that induces the development of new blood vessels, a pivotal mechanism for the proliferation of tumors, as it ensures an adequate blood supply. ERK, part of the MAPK signaling pathway, is often activated in response to growth factor signaling, including VEGF. When ERK is activated, it can contribute to cell proliferation, migration, and survival, further fueling the tumor’s expansion. Unlike the first pathway, this one is characterized by sustained activation, and the cells never return to their normal state, making it a hallmark of aggressive and invasive cancers.

The observation that VEGF activates ERK and influences GLUT-1 highlights a critical intersection between angiogenesis and cancer metabolism. VEGF expression levels should be regulated and maintained at a low threshold by normal physiological conditions. This mostly occurs through the regulatory influence of the hypoxia-inducible factor (HIF). Maintaining equilibrium in VEGF expression is highlighted since a level of VEGF can result in unregulated angiogenesis, a characteristic feature observed in numerous solid tumors. Simultaneously, the downstream activation of extracellular signal-regulated kinase (ERK) can extend the duration of cancer cell proliferation and migration. Within the context of this intricate biological system, several key regulatory entities, including VEGF, ERK, and OGT, exhibit a notable upregulation, with each of them playing significant roles in cancer progression, angiogenesis, and cell proliferation. These heightened expressions are central to the dynamics of this system.

The Qualitative model of this system unveils insightful trajectories. In particular, it highlights that a sustained upregulation of these three pivotal genes, namely, p53, β-catenin, and AKT, ultimately leads to a restorative cascade of events, including the return of oscillations within these genes and a gradual progression towards a state of recovery or normal homeostasis. In Figure 5, these trajectories are vividly depicted, showcasing the journey from a perturbed biological state to a normalized one. This visual representation provides a clear and intuitive understanding of the process. Furthermore, Figures 6, 7 provide a comprehensive depiction of the dynamic changes in gene expression along the important pathways throughout the transition of the biological system from one qualitative state to another. These figures essentially serve as maps, charting the changes in gene expression throughout the system’s journey. The key insight derived from these findings is that the biological system evolves from a qualitative, perturbed state to a cyclic, oscillatory state under the influence of calculated logical parameters. This transformation represents a pivotal attractor within the system, signifying a return to normal homeostatic conditions. It reveals the system’s intrinsic capacity to regain equilibrium, a fundamental element in the body’s response to maintain physiological balance and health. This insight carries significant implications for understanding and potentially manipulating the dynamics of biological systems, especially in the context of disease and therapeutic interventions.

**FIGURE 8:**
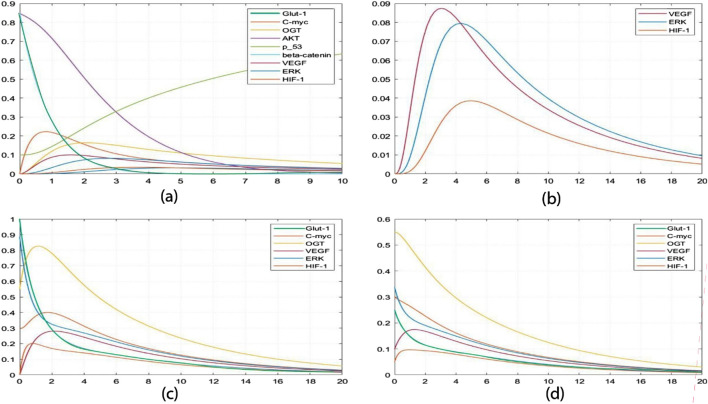
Simulation of the dynamic response of a cellular system: **(A)** It shows perturbations in p53, β-catenin, and AKT levels, with all other entities starting at zero concentration. **(B)** This graph shows the average value of VEGF, ERK, and HIF-1 over time. **(C)** It shows the initial high concentration of GLUT-1 and ERK, followed by a decrease in concentration in **(D)**. This decrease in concentration inhibits the concentration of other entities, such as OGT, HIF-1, and C-MYC.

Furthermore, SimBiology-generated concentration analysis graphs (Figure 8) enable precise tracking of minute changes in biological entity concentrations over time, down to seconds. Supplementary Material S5 provides the ordinary differential equations (ODEs) representing model interactions. The initial high concentrations of p53, β-catenin, and AKT depicted in Graph a (Figure 8) unveil a dynamic scenario. Analysis reveals that the p53 tumor suppressor experiences a concentration increase within one to three time units when interacting with AKT at a concentration of 0.31 mM/L. The observed increase in p53 concentration following its interaction with AKT suggests a critical role for p53 as a tumor suppressor. This dynamic indicates a responsive regulatory mechanism where AKT, a key signaling molecule in cell survival and proliferation, influences p53 levels to maintain cellular homeostasis and prevent uncontrolled cell growth. Simultaneously, β-catenin undergoes a decline from 0.8 mM/L to 0.02 mM/L, while AKT stabilizes at 0.03 mM/L, indicating a regulated biological response. The marked decline of β-catenin from 0.8 mM/L to 0.02 mM/L indicates a potential suppression of its oncogenic functions. β-catenin is involved in cell adhesion and signaling pathways that promote proliferation. Its decrease, alongside increased p53 levels, hints at a coordinated response to mitigate cancer progression. Initially, concentrations of C-MYC and VEGF surge significantly, reaching approximately 0-0.21 mM/L. The significant increase in C-MYC and VEGF concentrations reflects heightened cellular activities such as proliferation and angiogenesis, processes crucial for tumor growth and metastasis. Elevated VEGF, in particular, drives the formation of new blood vessels, supplying nutrients and oxygen to rapidly growing tumors. This surge signals heightened cell proliferation, angiogenesis, and potential alterations in cellular adhesion influenced by OGT. This coordinated biological activity enhances the invasive potential of cancer cells, facilitating metastasis to distant organs. Notably, this heightened state transitions into a stable equilibrium alongside critical entities such as HIF-1, ERK, and GLUT-1. The transition to a stable equilibrium involving HIF-1, ERK, and GLUT-1 suggests an integrated network where these factors collaboratively enhance tumor aggressiveness. HIF-1, known for its role in cellular response to hypoxia, promotes angiogenesis and metabolic adaptation, while ERK signaling is central to cell proliferation. GLUT-1, a glucose transporter, facilitates metabolic shifts necessary for the increased energy demands of cancer cells. Moving to Graph b (Figure 8), simulations monitoring VEGF, ERK, and HIF-1 concentrations over 20 seconds unfold. VEGF concentrations peak at 0.087 mM/L, while ERK concentrations approach 0.08 mM/L within five to six time units. The observed peak concentrations of VEGF and ERK correlate with enhanced angiogenesis and metabolic reprogramming. This interplay between signaling pathways and nutrient uptake is critical for sustaining tumor growth and supporting the invasive potential of cancer cells. The distinctive downregulation of VEGF compared to the slower degradation of ERK suggests an intricate regulatory mechanism to maintain homeostasis within the tumor microenvironment. This insight emphasizes the potential for therapeutic strategies that focus on modulating these dynamics to prevent excessive activation of pro-tumorigenic pathways. HIF-1 concentrations reach a maximum of 0.038 mM/L around the 5-time unit mark but regress to near zero by the 20-time unit mark (0.002 mM/L). Building upon insights from Figure 6, it becomes evident that VEGF stimulates the expression of HIF-1 by increasing ERK concentrations, thereby influencing angiogenesis and tumor progression. Graph c (Figure 8) introduces an intriguing perspective on hyper activation of ERK and GLUT-1. Elevating ERK concentrations (0.9 mM/L) influences GLUT-1 (1.0 mM/L), underscoring the critical intersection between angiogenesis and cancer metabolism. Consequently, OGT concentrations escalate to 0.82 mM/L, alongside heightened HIF-1 (0.2 mM/L) and VEGF (0.88 mM/L) within approximately two-time units, propelling the system toward a more aggressive and cancerous state. In Graph d (Figure 8), inhibiting the concentrations of ERK (0.25 mM/L) and GLUT-1 (0.35 mM/L) restrains OGT, VEGF, and HIF-1, placing the system in an intermediate state. This strategic intervention prevents the system from progressing toward hyper activation, offering potential insights into therapeutic interventions to mitigate aggressive cancer states. The observed pairwise concentration dynamics reveal a distinctive pattern: VEGF undergoes more rapid downregulation than ERK degradation, suggesting a regulatory mechanism for maintaining homeostasis. This implies that therapeutic strategies should prioritize expeditious clearance of VEGF post-function and a deliberately slower degradation rate for ERK, mitigating the risk of prolonged HIF-1 activation and downstream signaling cascade hyperactivation. The findings highlight the importance of targeting specific pathways. For instance, strategies to promote the rapid clearance of VEGF post-activation could limit angiogenesis, while carefully controlling ERK degradation may prevent prolonged HIF-1 activation. This dual approach could reduce the risk of enhancing tumor aggressiveness. Another critical insight from this constraint relationship is the necessity for GLUT-1 overexpression under cancerous conditions to potentiate its activating effect on HIF-1, preventing premature OGT degradation. These unique insights highlight the importance of thorough wet-lab explorations into the functions of HIF-1, VEGF, OGT, ERK, and GLUT-1, especially in the context of targeted interventions designed to impede cancer cell proliferation.

## 4 Discussion

Over the last few decades, there has been an extensive amount of research that has contributed to a better understanding of glucose metabolism in both healthy and rapidly dividing cancer cells. HIF-1, VEGF and GLUT-1 have been identified as key contributors in the metabolic context, and their aberrant regulation is strongly linked to the onset and progression of diverse malignancies (Maxwell et al., 1997), (Pouysségur et al., 2006), (Wiesener et al., 2003). To unravel the complex dynamics of this regulatory network, a discrete model (0,1) was constructed, providing insights into the qualitative behaviors of these crucial entities (Bernot et al., 2004).

The qualitative model has uncovered insights into the intricate interplay between genes and proteins driving cancer progression. It predicts the presence of cycles representing homeostasis and a stable state indicative of disease. According to the most physiologically realistic approach, it is required that the levels of expression for all entities, except p53, β-catenin, and AKT, would exhibit oscillatory behavior in order to sustain a state of equilibrium, and constant expression is vital for sustaining homeostasis. Our proposed qualitative model comprehensively explores the complex dynamics within biological systems, focusing on the dynamics of genes and proteins in cancer progression. Analyzing state transitions and critical trajectories unveils pivotal factors driving cancer’s advancement and identifies potential therapeutic targets to disrupt this process. The study highlights three distinct trajectories explained in previously highest betweenness centrality path section, each with unique implications for cancer. The first pathway in Figure 6, characterized by high GLUT-1 and OGT expression, reflects the Warburg effect, indicating metabolic changes that fuel rapid cancer cell growth (Jóźwiak et al., 2014). A “deadlock” condition blocks the cells' ability to revert to a non-malignant form within this biological process. In contrast, the second pathway in Figure 7, characterized by increased levels of VEGF and ERK expression, plays a key function in the process of angiogenesis and the advancement of tumors, ultimately resulting in the development of aggressive forms of cancer (Wiesener et al., 2003), (Lee et al., 2006). Maintaining VEGF expression balance is crucial, as excessive VEGF can lead to uncontrolled angiogenesis, a common feature in solid tumors. This study emphasizes the importance of regulating key genes such as p53, β-catenin, and AKT to guide the biological system back to recovery and normal homeostasis. The illustrations of transitions and gene expression change along these critical paths provide valuable insights into the system’s journey. This research reveals the system’s innate ability to evolve from a perturbed state to a cyclic, oscillatory state, signifying a return to normal physiological conditions. This insight carries broad implications for understanding and potentially manipulating the dynamics of biological systems, particularly in the context of diseases and therapeutic interventions.

While the BRN approach used in this study offers valuable insights into the qualitative dynamics of cancer progression, it is important to recognize its inherent limitations that could affect the model’s accuracy and predictive power. The primary limitation of the BRN approach is its reliance on binary states (0,1), which oversimplifies the continuous and dynamic nature of gene and protein expression in biological systems, where responses often involve graded variations rather than sharp transitions (Banf and Rhee, 2017). This binary simplification may overlook subtle variations in gene expression and regulatory interactions that are crucial for a comprehensive understanding of the system’s complexity. Additionally, the model’s focus on a limited number of key players, such as HIF-1, VEGF, Glut-1, and p53, can restrict its scope, potentially excluding significant regulatory factors and interactions that play vital roles in cancer progression. To tackle these limitations, future discussions should emphasize integrating more sophisticated modeling techniques that accommodate continuous variables, as well as expanding the network to include a broader array of molecular players and their interactions. This approach could enhance the model’s accuracy and provide a more holistic view of the complex dynamics involved in cancer biology. However, while these findings offer a detailed view of the system’s regulatory behavior, the reliance on computational predictions warrants further experimental validation to strengthen the real-world applicability of the results.

Future validation methods could include *in vitro* experiments involving cancer cell lines or *in vivo* animal models (Sun et al., 2018). For instance, cell culture models could be used to test the predicted oscillatory expression patterns of key genes such as HIF-1, VEGF, and Glut-1 under varying conditions that simulate cancerous growth. Additionally, animal models could help assess the role of VEGF in tumor angiogenesis and explore how therapeutic interventions targeting these pathways influence tumor development. This would allow researchers to directly test the model’s predictions, such as the impact of sustained VEGF and ERK expression on aggressive tumor growth or the role of Glut-1 and OGT in metabolic shifts that support cancer proliferation. Moreover, the SimBiology simulation revealed intricate molecular concentration dynamics over time, highlighting critical interactions. Key findings include the time-dependent relationships among p53, AKT, beta-catenin, C-MYC, OGT, VEGF, HIF-1, ERK, and GLUT-1, impacting angiogenesis and cancer metabolism. The study underscores the importance of targeted therapeutics, emphasizing faster VEGF downregulation and controlled ERK degradation to prevent hyper-activation of downstream signaling cascades and suggests further wet-lab exploration for effective cancer cell proliferation targeting. Overall, this study deepens our comprehension of the intricate dynamics of cancer progression and lays the foundation for developing targeted therapeutic strategies to restore homeostasis and combat the disease. However, to fully leverage these insights and develop practical applications, further wet-lab experiments are essential. Future experimental work could not only validate the predicted interactions but also guide the development of novel therapeutic strategies aimed at disrupting the key pathways identified in this model. For instance, by experimentally targeting the pathways related to VEGF, Glut-1, and p53, it may be possible to design treatments that halt tumor growth by restoring the system to a balanced, oscillatory state that resembles normal physiological conditions. It brings us closer to deciphering the complexity of cancer and offers hope for more effective interventions in the future.

## 5 Conclusion

This study offers significant insights into the complex regulatory networks that govern cancer progression, particularly through the lens of key pathways such as PI3K-Akt, HIF-1, and Wnt signaling. By leveraging bifurcation analysis and trajectory mapping, the model identifies crucial decision-making points in cellular processes that dictate whether a system moves toward a normal state, enters a quiescent phase, or spirals into a pathological, cancerous deadlock. Key components like AKT, HIF-1, VEGF, and GLUT-1 emerge as pivotal regulators within this framework. Their dynamic interplay highlights how external factors such as oxygen availability, metabolic stress, and growth signals drive either adaptive responses or malignant transformations. The model shows that persistent activation of these pathways can trap the system in an oncogenic state, suggesting that timely intervention at these critical points could help restore normal cellular function.

Additionally, this analysis provides insights into specific regulatory mechanisms, such as the role of HIF-1, VEGF and β-catenin in listing the balance between angiogenesis and tumor inactivity. The model identifies conditions under which the system may recover from cancerous states, emphasizing the importance of targeting these molecular drivers therapeutically. By identifying logical control parameters that either promote recovery or push the system toward malignancy, the model not only deepens our understanding of cancer biology but also opens new avenues for therapeutic innovation. Overall, this research contributes to a broader understanding of cancer as a dynamic, non-linear process, where small perturbations in key pathways can lead to vastly different outcomes. This enhanced knowledge of state transitions and critical thresholds offers a promising framework for future cancer therapies that aim to disrupt these pathways at key intervention points, thereby preventing cancer progression or promoting recovery from malignant states.

## Data Availability

The original contributions presented in the study are included in the article/Supplementary Material, further inquiries can be directed to the corresponding author.
