# Supplementary material for: Dynamics simulations of hypoxia inducible factor-1 regulatory network in cancer using formal verification techniques
Source: Front Mol Biosci. 2024 Nov 7;11:1386930. doi: 10.3389/fmolb.2024.1386930 (PMC11599740; doi:10.3389/fmolb.2024.1386930)

Supplementary Material

**Dynamics Simulations of Hypoxia Inducible Factor-1(HIF-1) Regulatory Network in Cancer Using Formal Verification Techniques**

Hafiz Muhammad Faraz Azhar^1^, Muhammad Tariq Saeed^2^, Ishrat Jabeen^3^*

School of Interdisciplinary Engineering & Sciences (SINES), National University of Science & Technology (NUST), Sector H-12, 44000, Islamabad, Pakistan

*Corresponding: ishrat.jabeen@sines.nust.edu.pk

**Experimental observations:**

| OGT | Under hypoxic condition OGT Upregulated and essential for tumor cell proliferation[1]. |
| --- | --- |
| C-myc | High level C-myc expression in hypoxia has been shown to drive vasculogenesis and promote metastasis[2][1]. |
| HIF-1 | HIF equitably regulates tissue oxygen supply and energetic metabolism and its expression is high[3][4][5]. |
| VEGF | Oxygen dispense inadequate stimulates angiogenesis through high regulation of VEGF[5][3]. |
| ERK | ERK phosphorylation to be upregulate under hypoxic conditions[6]. |
| Glut1 | Under hypoxic condition Glut1 expression levels is high in hepatocellular cancer cells (HCC)[7]. |
| .β-catenin | The upregulation of β-catenin in the cytosol initiate the expression of target gene such as c-myc[8]. |
| P53 | The increased level of p53 protein during hypoxic condition is due to a abruptly stabilized by cellular stresses and which has a major role in the cell responses[9]. |
| AKT | The phosphorylation of Akt is dramatically decreased under hypoxic environment[10]. |

**References:**

[1] Y. Lei *et al.*, “O-GlcNAcylation of PFKFB3 is required for tumor cell proliferation under hypoxia,” *Oncog. 2020 92*, vol. 9, no. 2, pp. 1–12, Feb. 2020, doi: 10.1038/s41389-020-0208-1.

[2] L. E. Huang, “Carrot and stick: HIF-α engages c-Myc in hypoxic adaptation,” *Cell Death Differ. 2008 154*, vol. 15, no. 4, pp. 672–677, Jan. 2008, doi: 10.1038/sj.cdd.4402302.

[3] K. S. Chae *et al.*, “Opposite functions of HIF-α isoforms in VEGF induction by TGF-β1 under non-hypoxic conditions,” *Oncogene 2011 3010*, vol. 30, no. 10, pp. 1213–1228, Nov. 2010, doi: 10.1038/onc.2010.498.

[4] R. Das *et al.*, “TGF-β2 is involved in the preservation of the chondrocyte phenotype under hypoxic conditions,” *Ann. Anat. - Anat. Anzeiger*, vol. 198, pp. 1–10, Mar. 2015, doi: 10.1016/J.AANAT.2014.11.003.

[5] E. M. Hendriksen *et al.*, “Angiogenesis, hypoxia and VEGF expression during tumour growth in a human xenograft tumour model,” *Microvasc. Res.*, vol. 77, no. 2, pp. 96–103, Mar. 2009, doi: 10.1016/J.MVR.2008.11.002.

[6] S. Y. Yoon *et al.*, “uPAR expression under hypoxic conditions depends on iNOS modulated ERK phosphorylation in the MDA-MB-231 breast carcinoma cell line,” *Cell Res. 2006 161*, vol. 16, no. 1, pp. 75–81, Jan. 2006, doi: 10.1038/sj.cr.7310010.

[7] T. Amann *et al.*, “GLUT1 Expression Is Increased in Hepatocellular Carcinoma and Promotes Tumorigenesis,” *Am. J. Pathol.*, vol. 174, no. 4, pp. 1544–1552, Apr. 2009, doi: 10.2353/AJPATH.2009.080596.

[8] X.-P. Cui, Y. Xing, J.-M. Chen, S.-W. Dong, D.-J. Ying, and D. T. Yew, “Wnt/beta-catenin is involved in the proliferation of hippocampal neural stem cells induced by hypoxia,” *Irish J. Med. Sci. 2010 1802*, vol. 180, no. 2, pp. 387–393, Sep. 2010, doi: 10.1007/S11845-010-0566-3.

[9] A. Sermeus and C. Michiels, “Reciprocal influence of the p53 and the hypoxic pathways,” *Cell Death Dis. 2011 25*, vol. 2, no. 5, pp. e164–e164, May 2011, doi: 10.1038/cddis.2011.48.

[10] D. Mottet *et al.*, “Regulation of Hypoxia-inducible Factor-1α Protein Level during Hypoxic Conditions by the Phosphatidylinositol 3-Kinase/Akt/Glycogen Synthase Kinase 3β Pathway in HepG2 Cells *,” *J. Biol. Chem.*, vol. 278, no. 33, pp. 31277–31285, Aug. 2003, doi: 10.1074/JBC.M300763200.

**SM Table 1:**

SMBioNet was used to estimate logical parameter values: The serial number of each parameter in the SMBioNet input file is listed in the first column (Sr) in order of appearance. The resources, acceptable expression levels, and each parameter are listed in the second, third, and fourth columns. The final values of the logical parameters that SMBioNet (Selected) computed are shown in the fifth column.


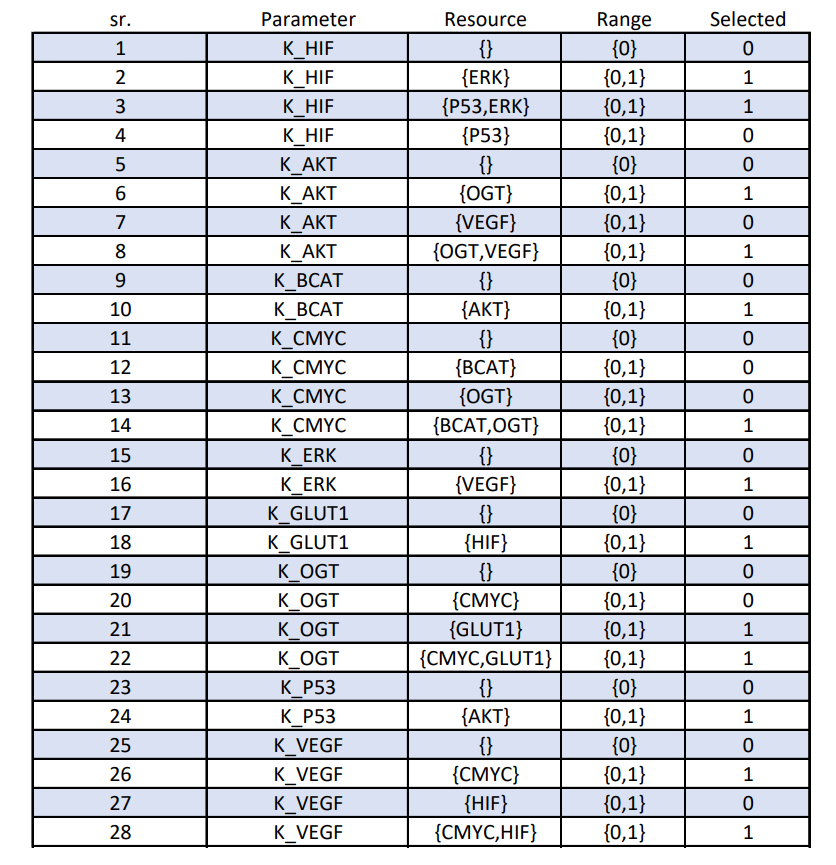

Supplement: Supplementary file 1 [file DataSheet1.zip › Supplementary Material/Supplementary Material S1.docx]
